# Bone Microarchitecture and Biomechanics of the Necrotic Femoral Head

**DOI:** 10.1038/s41598-017-13643-2

**Published:** 2017-10-17

**Authors:** Jian-xiong Ma, Wei-wei He, Jie Zhao, Ming-jie Kuang, Hao-hao Bai, Lei Sun, Bin Lu, Ai-xian Tian, Ying Wang, Ben-chao Dong, Yan Wang, Xin-long Ma

**Affiliations:** 1 Orthopaedics Institute, Tianjin Hospital, Tianjin, 300050 People’s Republic of China; 2 Tianjin Hospital, Tianjin University, Tianjin, 300211 People’s Republic of China; 3 Biomechanics Labs of Orthopaedics Institute, Tianjin Hospital, Tianjin, 300050 People’s Republic of China

## Abstract

The mechanism behind osteonecrosis of the femoral head (ONFH) remains unclear. The aim of this study was to explore the pathogenesis of ONFH from a biomechanical standpoint to provide a theoretical basis for improved treatments. We compared the bone structure of fractured femoral heads with that of necrotic femoral heads by Micro-CT scanning and histological evaluation. In addition, we compared the biomechanical properties of each zone in fractured femoral heads with those in necrotic femoral heads by using biomechanical tests. Compared with fractured femoral heads, bone microarchitecture and bone morphometry in necrotic zone and sclerotic zone of necrotic femoral heads have altered markedly. In addition, the biomechanical properties of the necrotic zone in femoral heads weaken markedly, while those of the sclerotic zone strengthen. We hypothesize that discordance between bone structure and function of the femoral head may be involved in the pathogenesis of ONFH and that more attention should be paid to the prevention and treatment of such discordance.

## Introduction

Osteonecrosis of the femoral head (ONFH) is a debilitating and intractable disease in orthopaedics. Its prevalence is increasing and commonly affects patients in the third, fourth, and fifth decades of life^1–4^. The natural progression of ONFH is rapid and can progress to femoral head collapse or secondary hip osteoarthritis within a few years^5,6^. The disease severely affects patients’ work and leisure activities, especially for young patients, and therefore, there is an urgent need to improve patient quality of life. Moreover, ONFH can cause significant economic and social burdens. Treatments for ONFH include non-surgical and surgical methods, although surgical treatment is the mainstream therapy. There are a variety of surgical treatments for ONFH, including core decompression, vascularized bone grafting, articulated hip distraction, osteotomy, hip resurfacing arthroplasty, and total hip arthroplasty^7^. While the efficacy of these treatments remains controversial, total hip arthroplasty provides excellent early pain relief and good functional outcomes for ONFH patients. However, total hip arthroplasty requires more host bone to be removed and, therefore, restricts future surgical options. In addition, poorer durability of prostheses related to other causes of joint degeneration has been observed in ONFH patients^1,7^. A lack of understanding regarding the pathogenesis of a disease can result in difficultly treating the condition. Risk factors for ONFH, such as corticosteroids, alcohol abuse, trauma, and sickle cell disease^8^, can be specified, but there remains no consistent conclusion regarding the pathogenesis of ONFH.

A series of hypotheses, such as blood flow disturbance^9^, osteocyte apoptosis, lipid metabolism disorder, gene polymorphisms, immune factors and biomechanical factors^10^, have been proposed. Many scholars consider interruption of the circulation to be responsible for the pathogenesis of ONFH^1,8^. Chandler called ONFH as “coronary disease of the hip”^11^ and the term “avascular necrosis of the femoral head” has been used in many studies^12,13^. Ueo *et al*.^10^ noted that the area of necrosis in the femoral head does not correspond to the vascular distribution and that biomechanical factors might play an important role in the development of ONFH. In the clinic, patients with traumatic ONFH usually develop symptoms 1 to 3 years after operation. If blood flow disturbance is the true pathological factor in ONFH, patients would be expected to develop symptoms much earlier. Harold Frost, who developed the Mechanostat, first proposed in 1961 that trabecular microfractures might be the cause of ONFH^14^. Yang *et al*.^15^ showed that collapse of necrotic femoral heads was caused by fatigue fractures. The hip joint acts as the major weight-bearing joint of humans, and a specific bone trabecular arrangement is the structural basis for the function of the femoral head. Changes in the internal space structure and biomechanical properties may play vital roles in the incidence and development of ONFH. Thus, we used micro-computed tomography (Micro-CT), hard tissue slicing, and biomechanical tests to evaluate changes in the internal space structure and biomechanical properties during ONFH. The aim of our study is to provide a better understanding of the pathogenesis of ONFH and potential clinical therapeutic targets.

## Results

**Figure 1:**
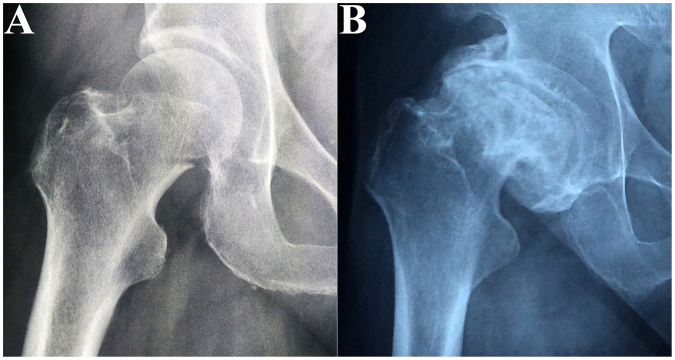
Anteroposterior X-ray of included patients. (**A**) Femoral neck fracture; (**B**) Femoral head necrosis.

We obtained 15 necrotic femoral heads and 15 fractured femoral heads from patients. The characteristics of the enrolled patients are presented in Table 1. The 15 patients with necrotic femoral heads were 50 to 67 years of age (58.4 ± 8.2 years), and the 15 patients with fractured femoral heads were 56 to 77 years of age (66.2 ± 9.3 years). Significant differences existed between the ages of the 2 groups (*P* < 0.05). Representative radiographs (Fig. 1) showed prominent femoral head collapse and femoral neck fracture. Ten of the femoral head necrosis cases were caused by corticosteroid intake, and the other five were caused by alcohol abuse. In addition, they were all in a late stage of the disease (FICAT III or IV stage). Micro-CT images and histological specimens showed that the position of the necrotic zone corresponded to that of the proximal compressive trabeculae zone, and the position of the sclerotic zone corresponded to that of the junctional zone. In addition, the positions of the distal compressive trabeculae zone and tensile trabeculae zone in the necrotic femoral head were the same as those in the fractured femoral head. Thus, we compared the necrotic zone of the diseased femoral head with the proximal compressive trabeculae zone of the fractured femoral head and compared the sclerotic zone of the diseased femoral head with the junctional zone of the fractured femoral head. The distal compressive trabeculae zone and tensile trabeculae zone were compared with the same parts in the fractured femoral head.

**Table 1:** Characteristics of patients in the 2 groups.

Group	Number	Gender	Age(years)	BMD(g/cm^2^)	Operative side		Garden		Ficat stage	
		(F/M)	Mean ± SD	Mean ± SD	Left	Right	III	IV	III	IV
FNF	15	10/5	66.2 ± 9.3	0.75 ± 0.13	8	7	5	10	—	—
ONFH	15	2	58.4 ± 8.2	0.71 ± 0.12^*^	10	5	—	—	7	8

Abbreviations: M = male, F = female, SD = standard deviation, BMD = bone mineral density, FNF = femoral neck fracture, ONFH = osteonecrosis of femoral head.

^*^Comparison with “FNF” group, P = 0.337.

### Space structure and bone morphometry

**Figure 2:**
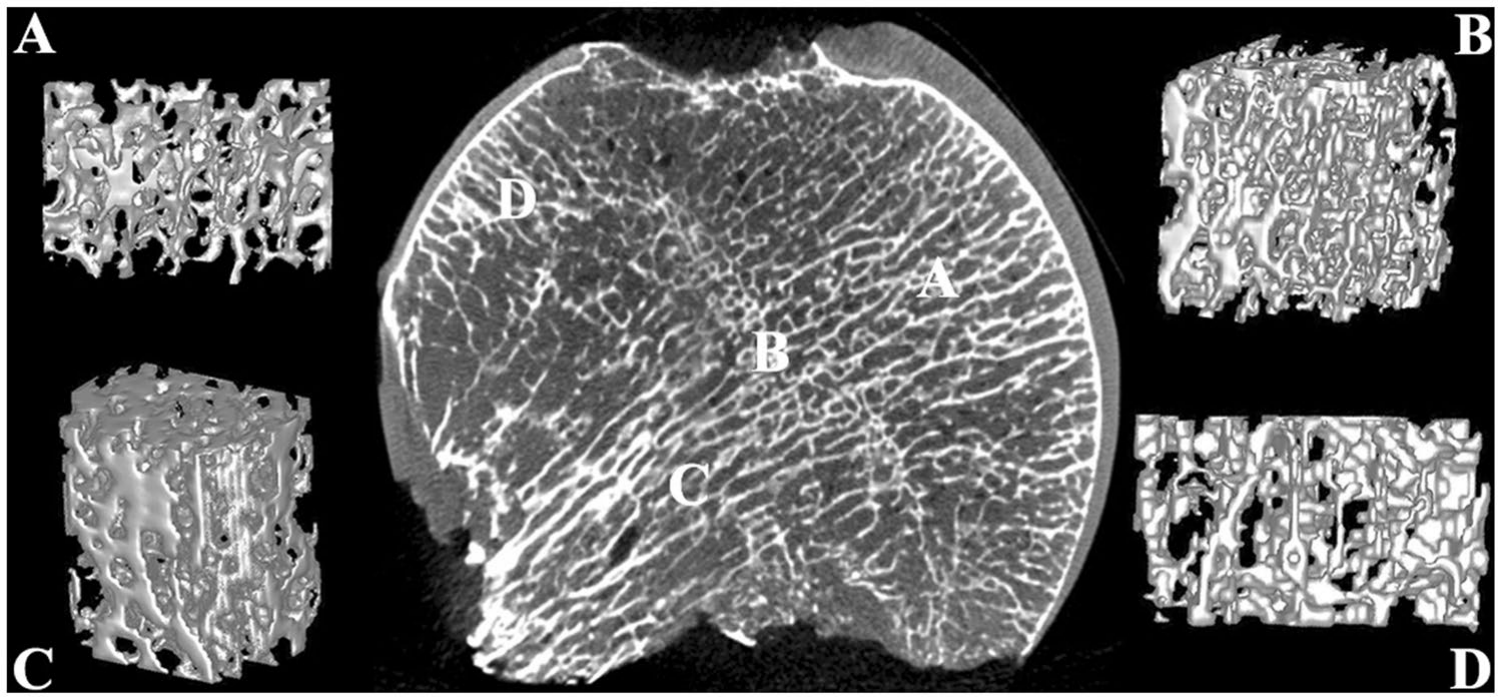
Coronal plane and reconstructed 3-D structure of the fractured femoral head. (**A**) Proximal compressive trabeculae zone; (**B**) Junctional zone; (**C**) Distal compressive trabeculae zone; (**D**) Tensile trabeculae zone.

The outline of the fractured femoral head in the coronal plane was smooth and approximately circular (Fig. 2), and the structure of the cartilage was intact and regular. The internal trabecular bone structure was largely intact and arranged in an orderly manner, and the arrangement of the trabecular bone underlying the cortical bone was nearly perpendicular to the cortical bone as a whole. We can see the reconstructed 3-D structure of each zone of a fractured femoral head in Fig. 2: the trabecular bone in each zone was arranged in an orderly and regular manner; the trabecular separation in each zone was consistent, and the trabecular separation in the tensile trabeculae zone appeared to be greater than in other zones (proximal compressive trabeculae zone, junctional zone, distal compressive trabeculae zone).

**Figure 3:**
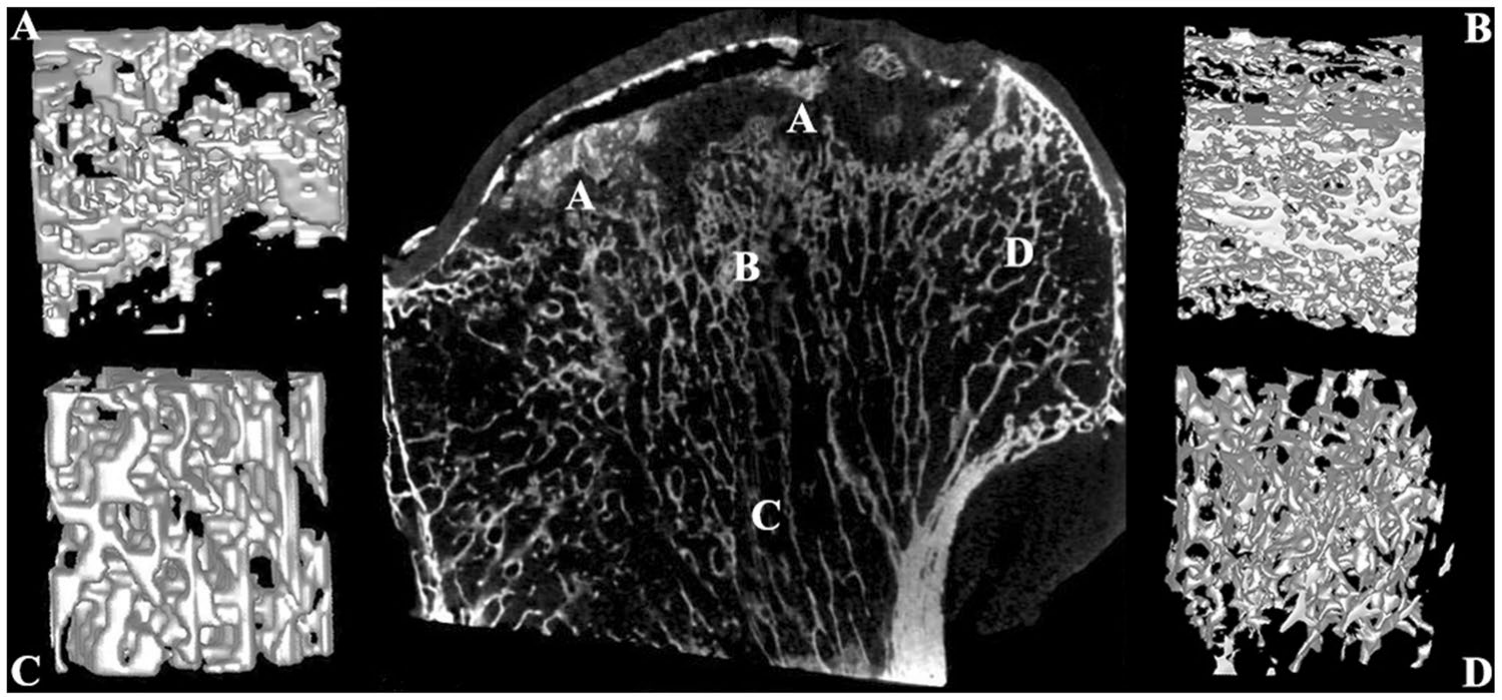
Coronal plane and reconstructed 3-D structure of the necrotic femoral head. (**A**) Necrotic zone; (**B**) Sclerotic zone; (**C**) Distal compressive trabeculae zone; (**D**) Tensile trabeculae zone.

In the necrotic femoral head, the smooth and approximately circular outline disappeared, and the head was deformed into various irregular shapes. Furthermore, the structure of cartilage was thinner or absent, and separation of cartilage from the subchondral bone was observed in 8 specimens. The inner trabecular bone structure and reconstructed 3-D structure of each zone of the necrotic femoral head (Fig. 3) showed that the trabecular bone in the necrotic zone was discrete, not intact, and some of the trabeculae were collapsed and squeezed. In addition, some trabeculae seemed to be resorbed and replaced by other non-bone tissues, and in the underlying sclerotic zone, the trabecular bone had a disorderly and anisotropic arrangement; moreover, the trabeculae had become thick and syncretic, and in the distal compressive trabeculae zone and tensile trabeculae zone, the arrangement and separation of the trabeculae were unchanged and remained uniform.

**Figure 4:**
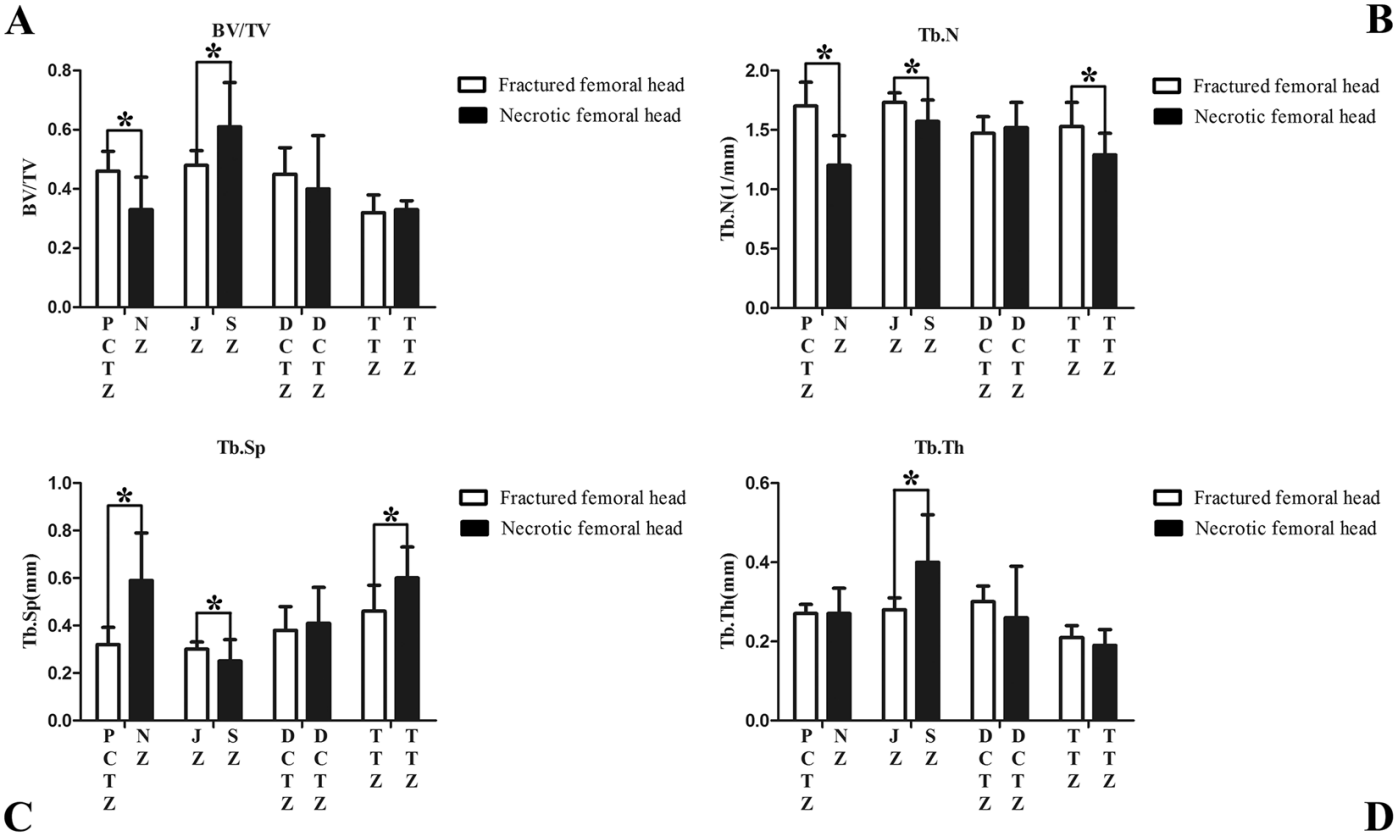
Results of bone morphometry of each zone in the fractured and necrotic femoral head. (**A**) Bone volume/total volume; (**B**) Trabecular number; (**C**) Trabecular separation; (**D**) Trabecular thickness. Data is presented as a mean ± standard deviation PCTZ = proximal compressive trabeculae zone, NZ = necrotic zone, JZ = junctional zone, SZ = sclerotic zone, DCTZ = distal compressive trabeculae zone, TTZ = tensile trabeculae zone; *P < 0.05.

The results of bone morphometry for each zone are shown in Table 2 and Fig. 4. In the fractured femoral head, the distal compressive trabeculae zone exhibited increased Tb.Th and Tb.Sp and decreased Tb.N compared to the proximal compressive trabeculae zone and junctional zone, while the tensile trabeculae zone exhibited lower values of BV/TV, Tb.N, and Tb.Th but higher values of Tb.Sp. Compared with the proximal compressive trabeculae zone in the fractured femoral head, the necrotic zone in the necrotic femoral head showed lower BV/TV and Tb.N (P = 0.001, P < 0.0001, respectively) and higher Tb.Sp (P = 0.0001). Compared to the junctional zone in the fractured femoral head, values of BV/TV and Tb.Th of the sclerotic zone increased (P = 0.003, P = 0.001, respectively), while Tb.N and Tb.Sp decreased (P = 0.002, P = 0.037, respectively). The distal compressive trabeculae zones of the fractured femoral head and necrotic femoral head showed similar bone morphometry. Compared with the tensile trabeculae zone of the fractured femoral head, that of the necrotic femoral head exhibited lower values of Tb.N (P = 0.003) and higher values of Tb.Sp (P = 0.003).

**Table 2:** Bone morphometry of each zone in fractured femoral head.

Zone Parameter	PCT	Junctional	DCT	TT
	Mean ± SD	Mean ± SD	Mean ± SD	Mean ± SD
BV/TV	0.46 ± 0.067	0.48 ± 0.05	0.45 ± 0.09	0.32 ± 0.06^*^
Tb.N (1/mm)	1.70 ± 0.20	1.73 ± 0.08	1.47 ± 0.14^*^	1.53 ± 0.20^*^
Tb.Sp (mm)	0.32 ± 0.072	0.30 ± 0.03	0.38 ± 0.10^*^	0.46 ± 0.11^*^
Tb.Th (mm)	0.27 ± 0.023	0.28 ± 0.03	0.30 ± 0.04^*^	0.21 ± 0.03^*^

Abbreviations: SD = standard deviation, PCT = proximal compressive trabeculae, DCT = distal compressive trabeculae, TT = tensile trabeculae.

^*^Comparison of proximal compressive trabeculae zone and junctional zone, p < 0.05.

### Histological evaluation

**Figure 5:**
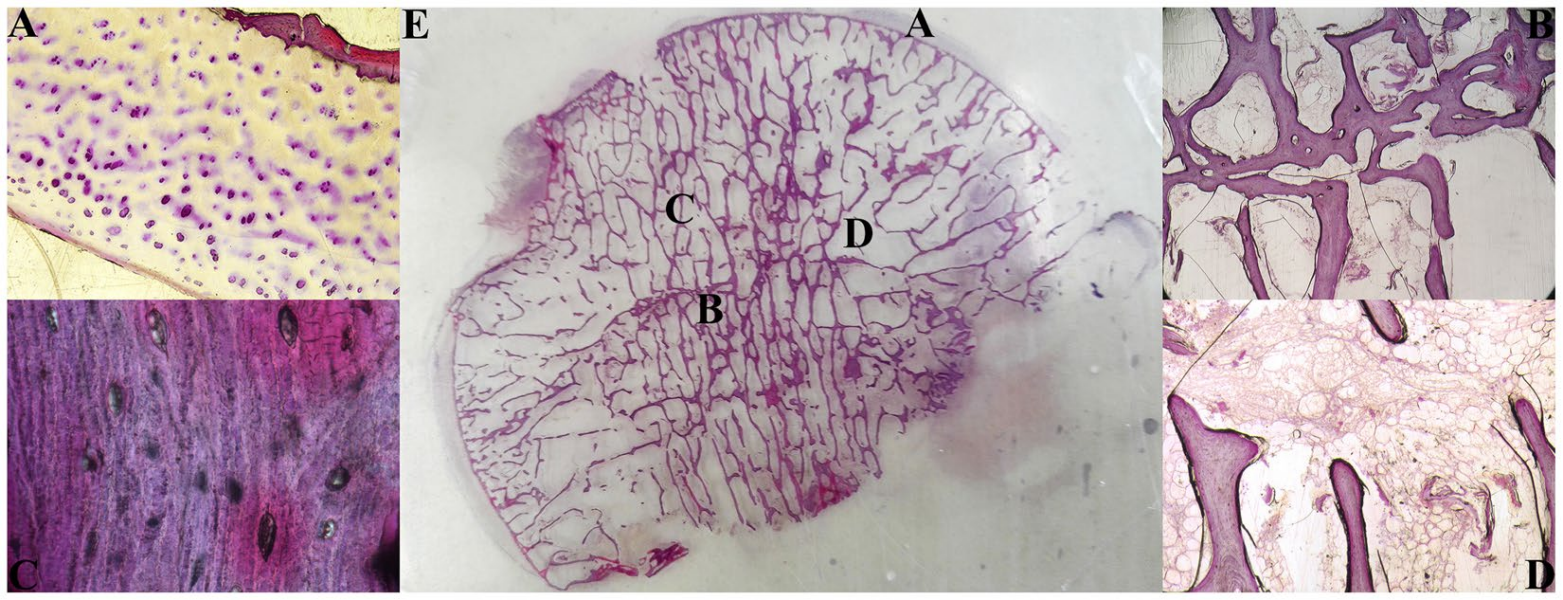
Hematoxylin and eosin staining of hard tissue slices for the fractured femoral head. (**A**) Cartilage (×40). (**B**) Trabeculae in junctional zone (×40). (**C**) Bone lacunae in trabeculae zone (×400). (**D**) Bone marrow (×40). (**E**) Hard tissue slice for fractured femoral head.

In the fractured femoral head, a uniform cartilage layer was observed, and the chondrocytes exhibited no necrocytosis. The subchondral bone plate was intact, and the bone trabeculae in each area had no fractures and an orderly arrangement. Osteocytes could be observed in most of the bone lacunae, and the proportion of empty lacunae in the fractured femoral head was significantly less than that in the necrotic femoral head (11.38% ± 1.8% VS 75.03% ± 8.98%, P < 0.05). In the bone marrow, the adipocytes were uniform in size (Fig. 5).

**Figure 6:**
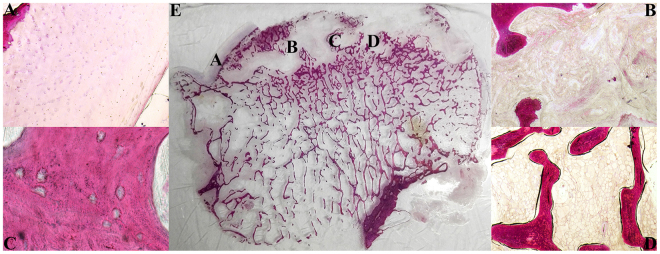
Hematoxylin and eosin staining of hard tissue slice for the necrotic femoral head. (**A**) Cartilage (×40); (**B**) Necrotic zone (×40); (**C**) Bone lacunae in trabeculae zone (×400); (**D**) Bone marrow (×40); (**E**) Hard tissue slice for fractured femoral head.

In the necrotic femoral head,the cartilage layer was irregular, cracked, or absent in some specimens. Chondrocytes appeared to have atrophied, and their number had decreased. Microfractures were observed at the subchondral bone plate in some specimens. Trabecular microfractures and breakage could be seen in the necrotic and sclerotic zones, and the arrangement of the bone trabeculae in the 2 zones was disorganised. In the necrotic zone, some of the bone trabeculae and bone marrow were replaced by granulation tissue or fibrous tissue, and neovascularisation could be observed in the granular or fibrous tissue. In the bone marrow, the proportion of haematopoietic tissue decreased. The number of adipocytes increased, and the size was not uniform (Fig. 6). In addition, the average size of adipocytes in the necrotic femoral head was larger than that in the fractured femoral head (79.15 ± 4.11 μm VS 66.21 ± 2.81 μm, P < 0.05).

### Biomechanical properties

**Figure 7:**
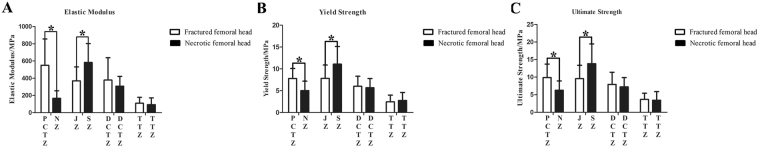
Results of biomechanical test of bone block in each zone. (**A**) Elastic modulus; (**B**) Yield strength; (**C**) Ultimate strength. Data is presented as a mean ± standard deviation. PCTZ = proximal compressive trabeculae zone, NZ = necrotic zone, JZ = junctional zone, SZ = sclerotic zone, DCTZ = distal compressive trabeculae zone, TTZ = tensile trabeculae zone; *P < 0.05.

Ninety-nine bone blocks were prepared from the fractured femoral head (29 in the proximal compressive trabeculae zone, 30 in the junctional zone, 25 in the distal compressive trabeculae zone and 15 in the tensile trabeculae zone), and 85 bone blocks were prepared from the necrotic femoral head (20 in the necrotic zone, 30 in the sclerotic zone, 20 in the distal compressive trabeculae zone and 15 in the tensile trabeculae zone). The ultimate strength, yield strength and elastic modulus of each zone and the statistical results are shown in Table 3 and Fig. 7. In the fractured femoral head, the tensile trabeculae zone exhibited weaker biomechanical properties compared with the compressive trabeculae zones (proximal compressive trabeculae zone, junctional zone, distal compressive trabeculae zone). The mean values of ultimate strength, yield strength and elastic modulus for the tensile trabeculae zone were all significantly smaller than those for the compressive trabeculae zones. Compared with the proximal compressive trabeculae zone in the fractured femoral head, the necrotic zone in the necrotic femoral head had a 70% lower elastic modulus (P = 0.001), a 36% lower yield strength (P = 0.021) and a 37% lower ultimate strength (P = 0.024). Compared with the junctional zone, the sclerotic zone had a 58% higher elastic modulus (P = 0.011), a 42% higher yield strength (P = 0.031) and a 45% higher ultimate strength (P = 0.037). For the biomechanical properties of the distal compressive trabeculae zone and tensile trabeculae zone, there were no significant differences between the necrotic femoral head and the fractured femoral head (P > 0.56 for all).

**Table 3:** Biomechanical properties of each zone in fractured femoral head.

Zone	PCT	Junctional	DCT	TT
Parameter	Mean ± SD	Mean ± SD	Mean ± SD	Mean ± SD
EM (MPa)	550.33 ± 306.31	369.23 ± 161.71	378.88 ± 258.63	109.57 ± 69.57^*^
YS (MPa)	7.79 ± 2.88	7.82 ± 3.06	6.00 ± 2.30	2.44 ± 1.55^*^
US (MPa)	9.88 ± 3.84	9.57 ± 3.82	7.93 ± 3.47	3.65 ± 1.78^*^

Abbreviations: SD = standard deviation, PCT = proximal compressive trabeculae, DCT = distal compressive trabeculae, TT = tensile trabeculae, EM = elastic modulus, YS = yield strength, US = ultimate strength.

^*^Comparison with all compressive trabeculae zones, p < 0.05.

## Discussion

Our study provides evidence that the bone microarchitecture and biomechanics of the femoral head are altered greatly during the late stages of ONFH. Compared with the fractured femoral head, the arrangement of the trabecular bone in both the necrotic and sclerotic zones of the necrotic femoral head was disorganised, and properties of the trabecular bone had changed markedly. The necrotic zone showed weaker biomechanical properties, while the sclerotic zone became stronger.

Structural degradation of cancellous bone has been suggested to have a strong effect on the onset of femoral head collapse^16^. Marked changes in bone microarchitecture have been observed in animal models^17,18^ and humans^19^, and we obtained similar results for both Micro-CT scans and histological evaluation. However, Wang C *et al*.^19^ did not detect any difference in micromechanical properties between the necrotic zone and the healthy zone. They compared the necrotic zone with another healthy zone in the same necrotic femoral head. In fact, we found some differences in bone morphometry and biomechanical properties between different zones of the same femoral head (Tables 2 and 3). Comparing different zones of the necrotic femoral head with corresponding zones in the fractured femoral head, we detected significantly different biomechanical properties. According to Wolff’s law^20^, bone in a healthy person or animal will adapt to the loads under which it is placed. If loading on a particular bone increases, the bone will remodel itself over time to become stronger to resist that sort of loading. The inverse is also true. The internal structure of bone is determined by its function, and in turn, the function of bone is influenced by its structure. Humans belong to the group of primates with bipedal locomotion, and loads equal to several times the body weight can act on the femoral head during routine activities. High compressive loading at the femoral head results in the complicated trabecular architecture found in the human femoral head, which includes the principal compressive group and the principal tensile group. In daily activities, most of the stress on the femoral head is borne by the compressive trabeculae; therefore, the trabeculae need to be compact and sufficiently strong. In contrast, the tensile trabeculae bear only a small part of the stress on the femoral head. Thus, these trabeculae do not need to be as strong, and both the mechanical quality and quantity of the trabeculae are low. Stress redistribution occurs in the initiation and progression of ONFH^21^. Stress concentrates at the interface of a necrotic zone and the underlying cancellous bone^10^. To adapt to the redistributed stress, the underlying cancellous bone is remodelled, during which the trabecular bone is thickened and has a disorderly and anisotropic arrangement, and its biomechanical properties are strengthened. Thus, the sclerotic zone is formed. Pascart T *et al*.^22^ detected a decrease in proteoglycan content in the sclerotic region, which was linked to an increase in bone remodelling. In addition, their histological examination showed the presence of woven bone corresponding to rapid disorganised new bone apposition. Bone remodelling and changes in bone structure are also related to other orthopaedic disorders; in osteoarthritis, subchondral bone loss, subchondral sclerosis, thickened subchondral bone plate, and increased trabecular thickness are observed^23^, and significantly altered structural parameters are also observed in osteoporosis^24,25^. The purpose of bone modelling and remodelling throughout life is to adapt the composition and structure of bone in relation to the prevailing loads^26^. Bone strength is influenced by many factors^27^; among these, microarchitecture is an important element of bone quality, and its integrity contributes to bone mechanical competence^28^.

We hypothesize that discordance of the internal structure and function of the femoral head may be a pathological factor in ONFH. For non-traumatic ONFH, risk factors such as glucocorticoid and alcohol abuse induce bone loss and weaken bone strength^29–31^. Continued stress on the femoral head and weakened bone strength lead to trabecular microfracture in a small primary area. The repair process is then activated, and imbalance can occur between repair and microfracture because of ongoing risk factors. Sustained stress on the femoral head can extend the area of microfracture leading to bone trabeculae collapse and the appearance of a necrotic zone. The collapsed trabeculae are resorbed and replaced by non-bone tissue, and thus, it will exhibit lower values of BV/TV, Tb.N, Tb.Th, higher values of Tb.Sp, and lower elastic modulus,yield strength and ultimate strength in the necrotic zone. Furthermore, stress shielding occurs at the border between the necrotic zone and the underlying trabeculae, and the magnitude and orientation of the stress is altered to adapt to this new stress. The underlying trabeculae are then remodelled and develop increased trabecular thickness, a disordered trabecular arrangement and strengthened biomechanical properties (increased values of BV/TV and Tb.Th, decreased Tb.Sp, and higher elastic modulus, yield strength and ultimate strength in the sclerotic zone). If a new mechanical equilibrium is achieved, the patient’s symptoms will improve (although the lower limb is shortened, pain will be relieved); otherwise, the collapse proceeds until a new mechanical equilibrium is achieved. For traumatic ONFH, although the femoral head is fixed by surgery, anatomical reduction is not always achieved, and the direction of the inner trabecular arrangement is no longer coordinated with the orientation of stress on the femoral head. Discordance of the internal structure and stress on the femoral head leads to trabecular microfracture in a small primary area, and the subsequent process is the same as for non-traumatic ONFH.

Therefore, preventing discordance of the internal structure and stress on the femoral head and improving bone strength may be effective for preventing and treating ONFH. Bisphosphonate zoledronic acid treatment has been shown to preserve femoral head architecture after traumatic ONFH in a rat model^32^, and clinical studies^33,34^ have confirmed the preventive effect of bisphosphonates on early collapse of the femoral head in ONFH. Tantalum combines strong mechanical resistance with high biocompatibility and osteoconductive and osteoinductive properties, and it has been used with the aim allow bone growth to occur while providing mechanical support^35,36^. Clinical studies^36–38^ and a meta-analysis^39^ regarding these treatments have shown appreciable improvement for patients with early stage ONFH. In addition, core decompression is commonly used in the early treatment of ONFH, the aim of which is to reduce bone marrow pressure as well as to increase blood flow to the femoral head^35^, although clinical outcomes are not as encouraging^40–42^. With regard to traumatic ONFH, the quality of reduction has a significant effect on the development of ONFH in patients with femoral neck fracture^43,44^; poor quality reduction may lead to more severe discordance within the internal structure and greater stress on the femoral head, and thus, the possibility of developing ONFH increases sharply.

Our study has several limitations. First, the indication of total hip arthroplasty for femoral neck fracture in our hospital signifies that the fractured femoral head specimens are from older patients. Age can influence the bone structure and biomechanical properties of the femoral head, although we did use ANCOVA to correct the age bias. Second, patients with ONFH who request total hip replacement treatment are always in an advanced stage of the disease. The necrotic femoral head specimens used in this study were all of stage FICAT III or IV. Because specimens at earlier stages were not obtained, their internal space structure and biomechanical properties remain unknown. Third, changes in bone space structure and biomechanical properties of the necrotic femoral head represent problems of cell biology, which we plan to explore in further studies.

## Conclusions

Discordance of the internal structure and function of the femoral head may be involved in the pathogenesis of ONFH, and we recommend that more attention is paid to the prevention and treatment of such discordance.

## Materials and Methods

### Materials

Human femoral heads were obtained from patients with femoral neck fracture or femoral head necrosis who underwent total hip arthroplasty in Tianjin Hospital from June 2016 to January 2017. The criteria for patients with femoral neck fracture were femoral neck fracture with an indication for total hip arthroplasty treatment and no history of congenital dysplasia of the hip, bone tumour, femoral head deformity or internal fixation affecting the structure of the femoral head. The criteria for patients with femoral head necrosis were femoral head necrosis with an indication for total hip arthroplasty treatment and no traumatic history of the hip joint. This study was approved by the Institutional Review Board of Tianjin Hospital. In addition, informed consent was obtained from each patient. The methods were carried out in accordance with relevant guidelines and regulations.

## Methods

### Micro-CT scan

Femoral heads were scanned with the Inveon^TM^ Micro-CT (Siemens, Berlin, Germany) at a voltage of 80 kV and a current of 500 µA. The femoral head was scanned at a spatial resolution of 36 µm. We then used an Inveon analysis workstation to reconstruct the 3-D structure of the femoral head. From the coronal plane of the femoral head, we subdivided the head into 4 zones for both fractured femoral heads (proximal compressive trabeculae zone, junctional zone, distal compressive trabeculae zone and tensile trabeculae zone, Fig. 2) and necrotic femoral heads (necrotic zone, sclerotic zone, distal compressive trabeculae zone and tensile trabeculae zone, Fig. 3). In fractured femoral heads, the principal compressive trabeculae, which extend from the medial cortex of the head into the femoral neck, intersect with the principal tensile trabeculae extending from the lateral margin of the greater trochanter to the inferior aspect below the fovea^45^, and we defined the intersecting zone as the junctional zone (Fig. 2B). We further defined the respective upper and underlying zones of the junctional zone in the principal compressive trabeculae as the proximal compressive trabeculae zone and the distal compressive trabeculae zone (Fig. 2A and C). In addition, the zone adjacent to the junctional zone in the principal tensile trabeculae that was closer to the fovea was defined as the tensile trabeculae zone (Fig. 2D). Because all enrolled patients with ONFH were in a late stage of the disease (FICAT III or IV stage), evident collapse was observed in these necrotic femoral heads. We defined the zone at which the femoral head had evidently collapsed as the necrotic zone (Fig. 3A). The zone underlying the necrotic zone, the threshold of which was higher than other zones, was defined as the sclerotic zone (Fig. 3B). A cylindrical region of interest (ROI) of the same size was selected from each zone of the femoral head. The trabeculae and bone marrow were separated by the threshold function^46^. The bone volume/total volume (BV/TV), trabecular thickness (Tb.Th), trabecular number (Tb.N) and trabecular separation (Tb.Sp) of each ROI were calculated^47^. The BV was calculated using tetrahedrons corresponding to the enclosed volume of the triangulated surface, the TV was the volume of the entire ROI, and the BV/TV represented the amount of bone mass in the ROI. The Tb.Th was determined by filling the structure with the maximal spheres using a distance transformation and calculating the mean thickness of the bone trabeculae. The Tb.Sp was calculated via the same procedure as used for the Tb.Th, but the voxels representing non-bone parts were filled with maximal spheres, allowing the thickness of the marrow cavities to be calculated. The Tb.N was taken as the inverse of the mean distance between the midaxes of the observed structure, which was the mean number of bone trabeculae in the ROI^47^.

### Histological evaluation

After Micro-CT scanning, the femoral head was sectioned coronally and then was divided into 3 parts; a 5-mm-thick histological section was obtained from the centre of the femoral head, which is the most representative area, while the other 2 parts were wrapped in saline-soaked gauze and frozen at −20 °C for later biomechanical tests^48^.

All histological sections were fixed in 10% neutral buffered formalin and dehydrated in different concentrations of alcohol (60%, 80%, 95%, 100%). The tissues were then embedded in Technovit 7200 VLC (Heraeus Kulzer, Wehrheim, Germany). All these calcified specimens were cut into 120-µm sections using the EXAKT-Cutting Grinding System (Norderstedt, Germany) and stained with haematoxylin and eosin (HE). All specimens were evaluated with a light microscope. The ratio of empty lacunae in the bone and the size of adipocytes in the bone marrow were calculated in four randomly selected fields^49^.

### Biomechanical test

**Figure 8:**
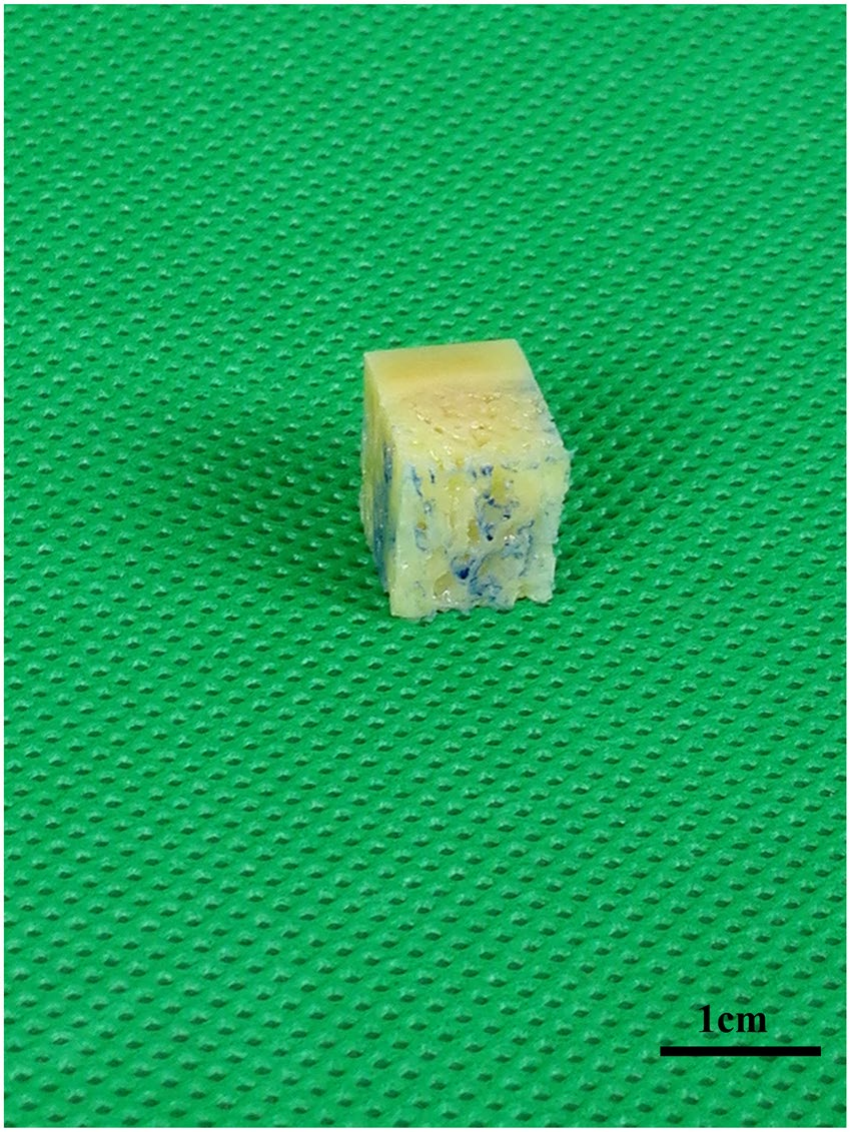
The cubic bone block.

**Figure 9:**
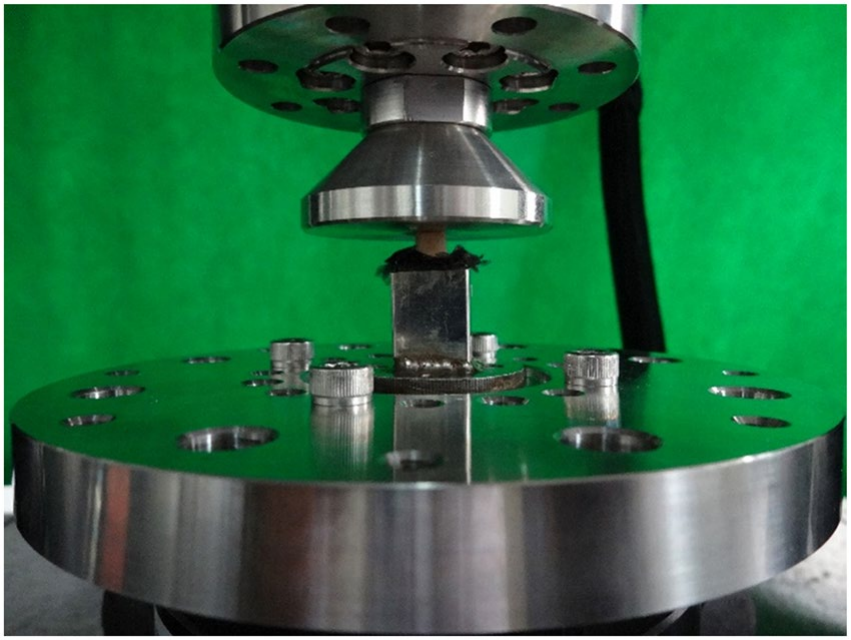
Biomechanical test of the bone block.

The sectioned femoral head parts frozen at −20 °C were thawed overnight at 6 °C prior to biomechanical tests^48^. A 1-cm^3^ cubic bone block (Fig. 8) was obtained from each zone of the femoral head using a high-speed, water-cooled EXAKT-Cutting Grinding System. To ensure the stability of the bone block during the biomechanical test, we fixed the bone block in a metal mould using polymethyl methacrylate. Bone blocks were tested to failure on a material testing machine (Bose 3510, America) by axial compression in displacement control at a rate of 0.016 mm/s (Fig. 9). Displacement and load were recorded 40 times per second. The ultimate strength, yield strength and elastic modulus were calculated to assess the biomechanical properties of the femoral head.

### Statistical analysis

All data are expressed as the mean ± SD. We used SPSS 19.0 software (IBM Corp., Armonk, NY, USA) to perform statistical analysis. The independent sample *t* tests were used to compare 2 groups, and ANOVA followed by the least significant difference (LSD) test was used for intra-group comparison. In addition, analysis of covariance (ANCOVA) was used to correct for age bias between patients with femoral head necrosis and femoral neck fracture. A value of P < 0.05 was considered statistically significant.

### Data availability

The authors declare that all data supporting the findings of this study are available in the article.

## Electronic supplementary material

supplemental information
