# Supplementary material for: Bone Microarchitecture and Biomechanics of the Necrotic Femoral Head
Source: Sci Rep. 2017 Oct 17;7:13345. doi: 10.1038/s41598-017-13643-2 (PMC5645321; doi:10.1038/s41598-017-13643-2)

Title

Bone Microarchitecture and Biomechanics of the Necrotic Femoral Head

Authors

Jian-xiong Ma[a,b,c,*], Wei-wei He[a,b,c,*], Jie Zhao[a,b,c], Ming-jie Kuang[a,b,c], Hao-hao Bai[a,b,c], Lei Sun[a,b], Bin Lu[a,b], Ai-xian Tian[a,b], Ying Wang[a,b], Ben-chao Dong[a,b], Yan Wang[a,b], Xin-long Ma[a,b]†

*These authors contributed equally to this work.

†Correspondence to Xin-long Ma.

[a] Orthopaedics Institute, Tianjin Hospital, Tianjin 300050, People's Republic of China

[b] Tianjin Hospital, Tianjin University, Tianjin 300211, People's Republic of China

[c] Biomechanics Labs of Orthopaedics Institute, Tianjin Hospital, Tianjin 300050, People's Republic of China

Name	Email
Jian-xiong Ma	mbiomechanics@126.com
Wei-wei He	2010021204@tmu.edu.cn
Jie Zhao	zhaoj_91@163.com
Hao-hao Bai	songbai1990@163.com
Ming-jie Kuang	doctorkmj@tmu.edu.cn
Lei Sun	sunleigys@163.com
Bin Lu	578794146@qq.com
Ai-xian Tian	tianax1986@126.com
Ying Wang	337533607@qq.com
Ben-chao Dong	441763510@qq.com
Yan Wang	280403251@qq.com

Corresponding author: Xin-long Ma; Postal address: NO.155, Munan Road, Heping District, Tianjin 300050, China; E-mail: maxinlong432@sina.com; Phone number: +86-15602179419

Trabecular microfractures in necrotic zone

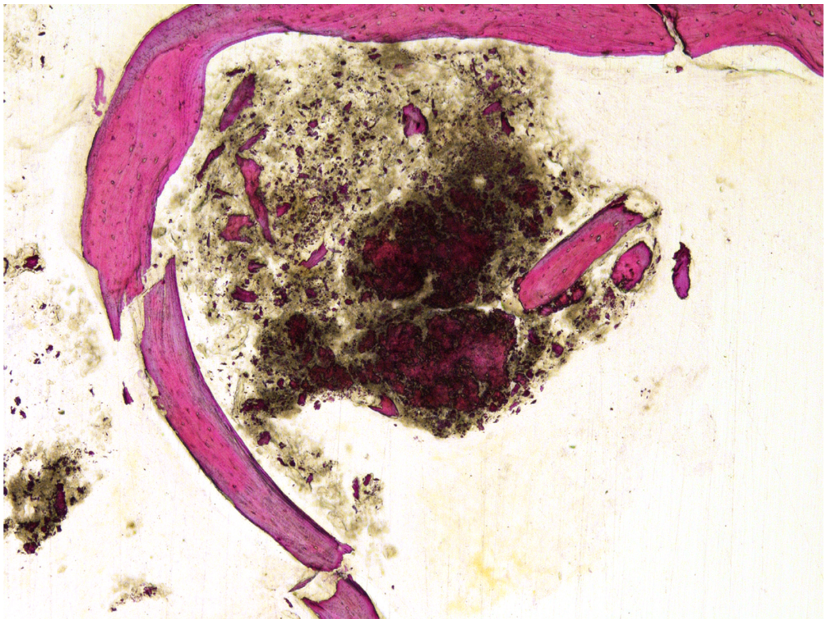

Trabecular microfractures in sclerotic zone

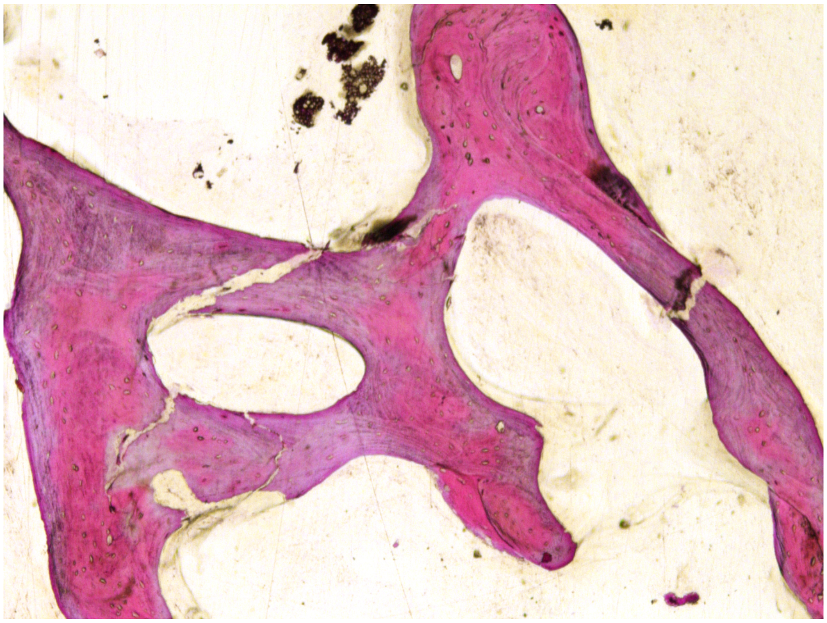

Supplement: Supplementary file 1 — supplemental information [file 41598_2017_13643_MOESM1_ESM.pdf]
